# Hosts and impacts of elongate hemlock scale (Hemiptera: Diaspididae): A critical review

**DOI:** 10.3389/finsc.2024.1356036

**Published:** 2024-02-12

**Authors:** Robert C. Venette, Angie Ambourn, Brian H. Aukema, Robert M. Jetton, Toby R. Petrice

**Affiliations:** ^1^ Northern Research Station, U.S. Department of Agriculture (USDA) Forest Service, St. Paul, MN, United States; ^2^ Plant Protection Division, Minnesota Department of Agriculture, St. Paul, MN, United States; ^3^ Department of Entomology, University of Minnesota, St. Paul, MN, United States; ^4^ Department of Forestry and Environmental Resources, North Carolina State University, Raleigh, NC, United States; ^5^ Northern Research Station, U.S. Department of Agriculture (USDA) Forest Service, East Lansing, MI, United States

**Keywords:** invasive alien species, *Tsuga*, *Abies*, plant-insect interactions, forest health

## Abstract

*Fiorinia externa* Ferris, elongate hemlock scale, was inadvertently introduced to North America from Japan. This insect is particularly problematic on hemlock, *Tsuga* spp., though it has been reported in association with several other conifers. The evidence that other conifers might be hosts, capable of supporting growing populations of the insect, has not been previously reviewed. Our review confirms that *F. externa* is an oligophagous pest of members of Pinaceae. Although species of Cupressaceae and Taxaceae have been reported as hosts of *F. externa*, they seem unable to support population growth of this pest. Evidence of the tree-killing potential of the insect, even on suitable hosts, is remarkably scant. The degree of pest risk posed by *F. externa* with respect to tree mortality in areas beyond the geographic range of hemlock seems modest, but uncertain.

## Introduction

**Figure 1:**
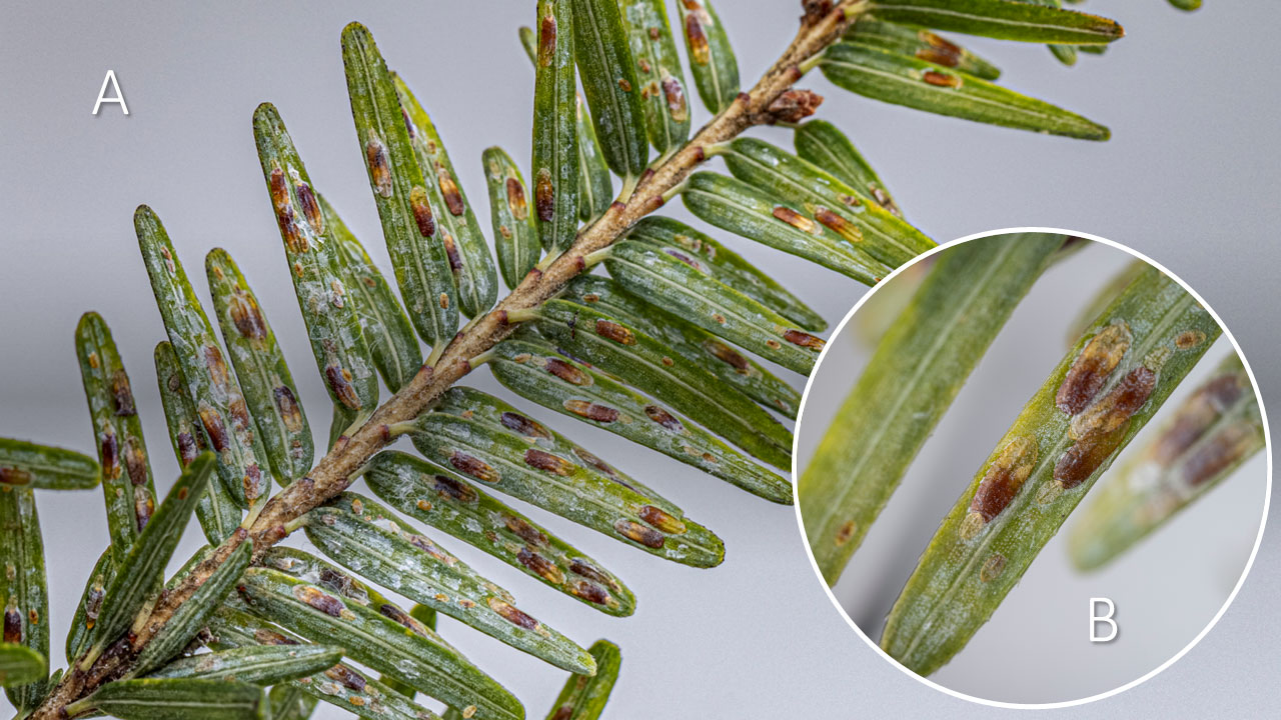
Elongate hemlock scale, *Fiorinia externa*, on the underside of eastern hemlock, *Tsuga canadensis*: **(A)** on the most current year of hemlock growth; and **(B)** closeup of adult females within tests. Both photographs by F.W. Ravlin, Department of Entomology, Michigan State University.

*Fiorinia externa* Ferris, elongate hemlock scale, is an alien armored scale (Hemiptera: Diaspididae) in North America. It is a primary threat to hemlock, *Tsuga* spp. (**Figure 1A**), that also are imperiled by the hemlock wooly adelgid, *Adelges tsugae* (Annand), another invasive alien insect (1). *Fiorinia externa* was first detected in 1908 in Queens and New York, NY, USA in association with an unspecified *Tsuga* sp. and Japanese hemlock, likely *Tsuga diversifolia* (Maxm.) Mast., respectively (2). By 1958, the insect had spread from New York state to Connecticut, Maryland, Massachusetts, New Jersey, Ohio, and Pennsylvania (3, 4), with no additional states reporting infestation through 1965 (5). By 1980, *F. externa* occurred as far south as Georgia (6). At about this time, the insect was spreading at 4.8 km/year in Connecticut (7). The insect is now established also in Delaware, Kentucky, Maine, Michigan, New Hampshire, North Carolina, Rhode Island, South Carolina, Tennessee, Vermont, Virginia, and West Virginia, states with significant acreages of Canadian/eastern hemlock, *Tsuga canadensis* (L.) Carrière (8). This insect has spread more slowly across the United States than many other invasive forest insects (9, 10).

At the time *F. externa* was first detected in the United States, the insect was recognized as having been accidentally imported from Japan (2). In his description of the species, Ferris (11) noted: “It is with regret that this species is described as new, since it would seem very probable that it has previously been named from some other part of the world.” Yet, no earlier descriptions of the species have been found. Ironically, confirmed records of the species outside the United States were unknown until Takagi (12) first definitively recovered and reported *F. externa* from Honshu and Shikoku islands of Japan and characterized Japan as the “cradleland” for the species. *Fiorinia externa* also has been reported from Fujian, Guangdong, and Sichuan, China (13, 14). Whether the species is native or introduced there is unclear. Van Driesche et al. (15) speculated that reports of *F. externa* from China might be misidentifications, as infested trees were difficult to locate. Elsewhere globally, *F. externa* was reported from an “alpine house” in England (16) but was never considered to be established in the wild (17). Kosztarab (18) also listed this scale as being present in Canada without details on the basis for this report. *Fiorinia externa* has not established in Canada, but future spread from the United States into Canada is probable (D. Pureswaran, personal communication).

Continued spread of *F. externa* in the United States is a concern to regulatory agencies, Christmas tree growers, and foresters (19–22). Human-mediated spread of this insect has been detected in California, Wisconsin, Minnesota, Oregon, and Florida on cut host material, e.g., Christmas trees, garlands, and wreaths, but the insect is not known to be established in these states (19, 21, 23, 24). The movement of infested nursery stock also may contribute to spread (25). Successful spread is contingent, in part, on the ability of the insect to locate and colonize hosts in its new environment(s) (9, 26). Herein, we briefly review the bionomics of *F. externa* that are relevant to interactions with conifers and summarize multiple studies on the diversity and quality of conifers as hosts. We conclude with a commentary on other taxa that might be hosts and the potential impacts this insect might have beyond the geographic range of hemlock.

## Bionomics

The biology of *F. externa* has been reviewed extensively elsewhere (e.g., 5, 27–30). The species has one to two generations annually with extensive overlap between generations due to a prolonged period of egg hatch (6, 28, 31, 32). Eggs and adult females are generally considered to be the primary overwintering stages. Adult females are pupillarial, having developed within the exuvium of the second instar (**Figure 1B**); the enclosure also is known as the test (33). Adults are dimorphic. Only males possess functional wings. The species reproduces sexually. Females mate shortly after completion of the third instar and contract to about one-third of their previous size within the test. Eggs are laid in two rows along the central mid-line within the space of the test. Because only adult males can fly, first instars (i.e., crawlers) are the dispersal stage for females, with movement over short distances achieved by walking and intermediate distances facilitated by wind (34). Colonization of isolated trees is happenstance, affected by the speed and direction of wind, the densities of crawlers on infested trees, and the proximity of source trees to uninfested trees (10). Longer dispersal distances might occur if crawlers could hitchhike on birds and mammals, but phoretic movement of *F. externa* has not yet been demonstrated.

*Fiorinia externa* is a piercing-sucking insect. This insect feeds on host plants through a feeding tube made from fused mandibular and maxillary stylets. A crawler creates a feeding site, typically on the underside of the newest needles and occasionally on the surface of developing cones (18), by scraping away the host epidermis with its forelegs and inserting the stylets between epidermal cells or into a stomate (5). The stylets remain inserted once feeding commences (5). The stylets penetrate mesophyll cells and allow the insect to extract photosynthates and other cell contents from the needles and to inject of phytotoxic saliva (29). For adult females, the stylet is 6-7 times the body length (5).

## Host range

In the first official U.S. report of the species, Sasscer (2) noted that the insect, then identified as *Fiorinia fiorinae japonica*, had been collected on *Tsuga* spp. He also shared anecdotal reports of the scale on other Pinaceae: Veitch fir, *Abies veitchi* Lindley; an unspecified *Pinus* sp.; a Japanese five-needled pine, *Pinus pentaphylla* Mayr [=*P. parviflora* var. *pentaphylla* (Mayr) Henry, currently]; Japanese black pine, *P. thunbergii* Parl.; and southern Japanese hemlock, *T. sieboldii* Carrière. He included additional hosts among Podocarpaceae: *Podocarpus elata* [=*P. elatus* R. Br. Ex Endl., currently], *P. chinensis* (Roxb.) Wall ex J. Forbes, and *P. nageia* R. Br. Ex Mirbel [=*Naegia nagi* (Thunb.) O. Kuntze, currently]. However, Sasscer (2) likely conflated potential hosts for *Fiorinia japonica* Kuwana, now known to have several hosts within Podocarpaceae, with hosts for *F. externa*, which has no confirmed hosts in that plant family (27).

The current list of hosts for *F. externa* was derived primarily from reported associations of the insect with different plants. Frequently, the reports summarized observations of plants growing near infested *Tsuga* spp. in ornamental plantings including arboreta. Some observations simply recorded the presence of *F. externa* on a plant. In other cases, observations included *F. externa* densities on different plants but often with no information about the size of the colonizing population or the time since infestation. If true, differences in densities of *F. externa* on different plants would reflect differences in host quality with respect to survivorship, developmental, or reproductive rates or chance variation in the number of crawlers that colonized a tree over time. In rare cases (e.g., 35), potential host trees were artificially infested to standardize initial colonization densities, so that differences in scale densities over time likely reflect differences in suitability among trees.

For this review, we assembled literature on potential hosts of *F. externa* published after Ferris’ (1942) description of the species (**Table 1**). Ferris (11) considered *Tsuga* spp. to be the primary hosts for *F. externa*. Thus, when possible, we compare the performance of *F. externa* on a plant relative to its performance on eastern/Canadian hemlock, *Tsuga canadensis* (L.) Carr. or Carolina hemlock, *T. caroliniana* Engelm. in the same study and location. ‘Performance’ is either the density of scales on individual trees (often expressed as scales/needle) or the proportion of trees that had been colonized. Qualitative ratings were assigned to facilitate comparisons among studies: ‘+++++,’ >80% performance as on a known host; ‘+++,’ ~40-60% performance as on a known host; ‘+,’ 1-20% performance as on a known host; and ‘-,’ not likely to be a host. In cases where non-host status may reflect chance escape from colonization, we use ‘-(?)’. Intermediate categories were applied as needed. Wallner (5) characterized *F. externa* densities as “heaviest”, “heavy”, and “light,” which we expressed as “+++++”, “++++”, and “+”, respectively. All studies in our review observed *F. externa* directly.

**Table 1:** Host status of conifers for *Fiorinia externa*.

Species	Common name	Host status	Source
Pinaceae			
*Abies alba*	Silver fir	+++	(35)
		++	(36)
*Abies amabilis*	Cascade fir	+++++	(35)
		+++++	(36)
*Abies balsamea*	Balsam fir	+	(35)
		Yes	(6)
*Abies cephalonica*	Greek fir	++++	(5)
		-(?)	(35)
*Abies concolor*	White fir	++++	(5)
		+++	(35)
		+	(36)
*Abies fargesii*	Farges fir	+++++	(35)
		+++++	(36)
*Abies fraseri*	Fraser fir	Yes	(6)
		+++++^*^	(37)
*Abies holophylla*	Manchurian fir	+	(35)
*Abies homolepis*	Nikko fir	-(?)	(35)
*Abies veitchii*	Veitch fir	+++++	(35)
		+++++	(36)
*Cedrus atlantica*	Atlas cedar	+++++	(35)
		++++	(36)
*Cedrus deodara*	Deodar cedar	-(?)	(35)
		++++^*^	(37)
*Cedrus libani*	Cedar of Lebanon	-(?)	(35)
*Picea abies*	Norway spruce	++++	(5)
		+	(35)
		+++++	(36)
*Picea asperata*	Dragon spruce	+	(35)
*Picea glauca*	White spruce	+	(35)
*Picea glehni*	Saghalin spruce	+	(35)
*Picea heterolepis*	Red twig dragon spruce	+	(35)
*Picea jezoensis*	Yezo spruce	+	(35)
*Picea koyamai*	Koyama spruce	+	(35)
*Picea mariana*	Black spruce	+	(35)
*Picea omorika*	Serbian spruce	+++	(35)
		+++	(36)
*Picea orientalis*	Oriental spruce	+	(35)
*Picea pungens*	Colorado blue spruce	++++	(5)
		+++++	(35)
		+++++	(36)
		++++^*^	(37)
*Picea smithiana*	Himalayan spruce	+	(35)
*Pinus aristata*	Rocky Mountain bristlecone pine	-(?)	(35)
*Pinus bungeana*	Lacebark pine	-(?)	(35)
*Pinus cembra*	Swiss stone pine	+	(35)
*Pinus clausa*	Sand pine	–	(37)
*Pinus densiflora*	Japanese red pine	-(?)	(35)
*Pinus elliotti*	Slash pine	+^n.s.^	(37)
*Pinus flexilis*	Limber pine	-(?)	(35)
*Pinus glabra*	Spruce pine	+^n.s.^	(37)
*Pinus griffithi*	Himalayan white pine	–	(35)
*Pinus lambertiana*	Sugar pine	–	(35)
*Pinus mugo*	Mugo pine	-(?)	(35)
*Pinus parviflora*	Japanese white pine	+	(35)
*Pinus pumila*	Dwarf stone pine	+	(35)
*Pinus resinosa*	Red pine	-(?)	(35)
*Pinus rigida*	Pitch pine	-(?)	(35)
*Pinus strobus*	Eastern white pine	+	(5)
		+	(35)
*Pinus sylvestris*	Scots pine	-(?)	(35)
*Pinus taeda*	Loblolly pine	++^*^	(37)
*Pinus virginiana*	Virginia pine	+++^*^	(37)
*Pseudotsuga menziesii*	Douglas fir	++++	(35)
		++	(36)
*Tsuga canadensis*	Eastern hemlock	+++++	(5)
		+++++	(35)
		+++++	(36)
		+++++/+++++	(38)
		+++++^*^	(37)
*Tsuga caroliniana*		+++++	(5)
		+++++	(35)
		+++++	(36)
		Yes	(6)
		+++++/+++++°	(38)
*Tsuga chinensis*		+++/+++++°	(38)
*Tsuga diversifolia*	Japanese hemlock	+++++	(5)
		+++++	(35)
		+++++	(36)
		n.a./+++++ °	(38)
*Tsuga heterophylla*		++/+++++°	(38)
*Tsuga mertensiana*		n.a./+++++ °	(38)
*Tsuga sieboldii*	Siebold hemlock	+++++	(35)
		++++	(36)
		n.a./+++++ °	(38)
Cupressaceae			
*Chamaecyparis thyoides*	Atlantic white cypress	–	(37)
*Cryptomeria japonica*	Japanese cedar	–	(35)
*Cupressus arizonica*	Arizona cypress	–	(37)
*Cupressus x leylandii*	Leyland cypress	–	(37)
*Juniperus virginiana* var *silicola*	Southern red cedar	+^n.s.^	(37)
*Thuja* x ‘Green Giant’	Arborvitae ‘Green Giant’	–	(37)
Taxaceae			
*Taxus baccata*	European yew	-(?)	(35)
*Taxus cuspidata*	Japanese yew	+	(5)
		-(?)	(35)
*Taxus foridana*	Florida yew	–	(37)
*Torreya taxifolia*	Florida torreya	+^n.s.^	(37)

Qualitative rankings describe the performance of scales on a conifer species relative to hemlocks (*Tsuga* spp.) in the same study: +,1-20%; ++, 21-40%; +++, 41-60%, ++++, 61-80%;+++++, >80% performance as on a known host; -, not likely to be host; and -(?), not likely to be a host or not colonized by chance.

*, significantly greater degree of infestation than a known non-host; ^n.s.^, degree of infestation not significantly different from a known non-host. °, as reported in Weston and Harper (38) and as observed in 2020 (Richard Weston, personal communication); n.a., not available.

### Pinaceae

*Fiorinia externa* is an oligophagous pest of Pinaceae, though not all species within this plant family are equally suitable. The insect is regularly characterized as a serious pest of hemlocks, especially eastern/Canadian hemlock, Japanese hemlock, and Carolina hemlock, particularly in ornamental plantings (3, 6, 31, 39–41). In Japan, the primary host appears to be southern Japanese hemlock, *Tsuga sieboldii* Carrière (12). In addition, early reports of *F. externa* from North America casually note its association with fir, *Abies* spp., and spruce, *Picea* spp., typically without reference to species (3, 39–41). Murakami (42) listed Sakhlin fir, *Abies sachalinensis* (F. Schmidt) Masters var ‘Mayriana’ as a host. Stimmel (6) observed *F. externa* on Fraser fir, *Abies fraseri* (Pursh) Poir. Williams (16) reported *F. externa* as persisting and likely reproducing on Korean fir, *Abies koreana* E.H. Wilson.

The highest quality hosts for *F. externa* include many hemlocks and firs (**Table 1**). Other high-quality hosts are Atlas cedar, *Cedrus atlantica* (Endl.) Manetti ex Carrière, Norway spruce, *Picea abies* (L) Karsten, and Colorado blue spruce, *Picea pungens* Engelmann. However, other spruces and cedars seem to be of substantially lower quality. Deodar cedar, *Cedrus deodara* (Roxb. ex D.Don) G.Don, and Douglas fir, *Pseudotsuga menziesii* (Mirbel) Franco, may be quality hosts for *F. externa*, but the performance of the insect on these hosts was highly variable among studies (**Table 1**). Pines, *Pinus* spp., tend to be intermediate- to low-quality hosts or non-hosts (**Table 1**).

### Cupressaceae

Members of this family are unlikely to be reproductive hosts (i.e., plant species that support population growth of a specified herbivore) for *F. externa*. Tao (14) lists the arborvitae *Biota orientalis* (L.) Endl [=*Platycladus orientalis* (L.) Franco, currently]; Chinese juniper, *Juniperus chinensis* L., and needle juniper, *Juniperus rigida* Siebold & Zucc. as hosts for *F. externa* without further information. No other sources independently corroborate these host associations. In earlier studies, false cypress, *Chamaecyparis* sp.; Japanese cedar, *Cryptomeria japonica* (L) Don, and junipers, *Juniperus* spp., growing next to infested hemlocks were not colonized by *F. externa* and therefore not considered to be hosts (35). Similarly, after being artificially infested on plants, *F. externa* failed to persist on the arborvitae *Thuja* x ‘Green Giant’ (a hybrid of *Thuja plicata* x *Thuja standishii*); Arizona cypress ‘Blue Ice’, *Cupressus arizonica* Greene; Atlantic whitecedar, *Chamaecyparis thyoides* (L.) B.S. P.; Leyland cypress, *Cupressus* x *leylandii* A. B. Jacks. & Dallim.; or southern eastern red cedar, *Juniperus virginiana* var *silicicola* (Small) E. Murray (37). *Fiorinia externa* began to feed on all of these plants except *Thuja* x ‘Green Giant’ and *Cupressus* x *leylandii*; typically, <10% of trees in Cupressaceae - but 57% of *J. virginiana* var *silicicola* – had live scales at 19 weeks after infestation (37). By 50 weeks after infestation, no Cupressaceae had live scales, except *J. virginiana* var *silicicola* whose 3% frequency of infestation was equivalent to a known non-host (37). Members of Cupressaceae have been confirmed as hosts of the congeneric *Fiorinia japonica* in multiple studies (27).

### Taxaceae

Members of this family are probably not reproductive hosts for *F. externa*. Yews, *Taxus* spp., were listed as (secondary) hosts in early publications about the species (3, 5, 30, 35, 39, 40). These reports typically noted the association of *F. externa* with the plant genus without mentioning species (3, 30, 39). Wallner (5) observed light densities of *F. externa* on Japanese yew, *Taxus cuspidata* Siebold et Zuccarini, compared to *Tsuga* spp. McClure and Fergione (35) documented that *F. externa* densities were less than one scale per 500 needles on European yew, *Taxus baccata* L, and Japanese yew. Dale et al. (37) recorded 90% of Florida torreya, *Torreya taxifolia* Arnott, and 80% of Florida yew, *Taxus floridana* Nuttall ex Chapman, were still infested at 19 weeks after artificial infestation; by 50 weeks, the frequency of infestation dropped to 3% and 0%, respectively, no different from known non-hosts.

## Impact

Feeding by *F. externa* can cause decline-like symptoms such as needle yellowing, needle cast, branch dieback, stunted growth, and occasionally tree death (4, 5, 39, 41). Feeding likely reduces overall host vigor and may increase tree susceptibility to other abiotic and biotic stresses. Unlike hemlock woolly adelgid, feeding by *F. externa* has no measurable effect on the phytohormones abscisic acid or salicylic acid (43). The ability of the insect to vector plant pathogens is unknown. Dense infestations give foliage a “whitewashed appearance” from the waxy secretions from male scales (3, 6, 40).

The potential for *F. externa* to kill trees, even of highly suitable hemlocks, remains unclear. The progression of plant symptoms first was described for ornamental trees as no natural stands or plant nurseries were known to be infested at that time (reviewed in (5)). McClure (44) and McClure and Fergione (35) reaffirmed *F. externa* as a potential tree-killing insect and casually expanded the scope of potential impacts to include forest hemlocks. These anecdotal descriptions were sufficient for Miller et al. (45) to list *F. externa* as a “serious pest” of hemlock, arguably among the more threatening species of non-native scale insects in North America. However, the frequency of mortality among infested trees or the extent of mortality at stand scales had/has never been reported for this insect. (Note: Zahradnik (46) did not include *F. externa* among “the more important or interesting” scales that affect conifers.) More recently, Mech et al. (47) concluded that *F. externa* is capable, at worst, of killing “individual healthy plants” and, thus, did not consider it to be a high-impact species.

The severity of symptoms is partially related to the density of *F. externa*. Densities of *F. externa* are affected by soil texture and moisture (44) and are positively correlated with the nitrogen content of needles, both within and among host species (48). McClure and Fergione (35) posited that a density of >6 scales/100 needles was sufficient to cause substantial hemlock-needle discoloration and drop from the lower crown, but they did not provide the basis for this threshold. At low densities (<1 scale/needle), *F. externa* did not affect growth or foliar nitrogen content of *T. canadensis*, but at similar densities, hemlock woolly adelgid occasionally did (49). The intensity, beyond being “high”, or duration of infestation that might be necessary for mortality has not been specified. McClure (50) argued that *F. externa* could kill hemlocks within 10 years if left uncontrolled but provided no data. Johnson and Lyon (51) suggested that infested hosts may begin to recover (i.e., flush new needles) if scale densities decline, for example, from intraspecific competition.

More recent research suggests that *F. externa* rarely kills trees and may prove modestly beneficial to hemlocks in the presence of hemlock woolly adelgid. During the late 1990s and early 2000s, the geographic range and density of *F. externa* substantially increased on Massachusetts and Connecticut (USA) hemlock with little apparent impact to tree health; hemlock mortality correlated more strongly with densities of hemlock woolly adelgid than *F. externa* (52). When hemlocks in this area were artificially infested with both insect species, densities of each insect were reduced by ~30%, but lateral growth was greater on trees infested with both species than with hemlock woolly adelgid alone (53). *Fiorinia externa* alone had no measurable impact on branch growth (53) or tree ring formation (54). Subsequent research has demonstrated that hemlocks with *F. externa* have similar foliar chemistry and growth rates as uninfested trees, unlike trees infested with hemlock woolly adelgid (49). The early presence of *F. externa* substantially lowered settlement rates for hemlock woolly adelgid crawlers (55); crawlers avoided settling at the base of needles where *F. externa* was already established (56). Ultimately, Miller-Pierce and Preisser (55) characterized *F. externa* as “relatively innocuous” to hemlock health in comparison to hemlock woolly adelgid. Preliminary observations from Michigan are consistent with these trends as no relationship was observed between current-year hemlock growth and *F. externa* densities (20).

## Discussion

*Fiorinia externa* is an oligophagous pest of trees in the Pinaceae. The phylogeny and evolutionary history of genera within this family (57) and patterns of host utilization by *F. externa* (**Table 1**) suggest other genera within Pinaceae might be heretofore unreported hosts, particularly *Keteleeria*, *Nothotsuga*, and *Pseudolarix*, all native to portions of eastern Asia. Further research into these potential host associations is warranted and might provide insights into the co-evolutionary history of *F. externa* with Pinaceae in Asia. Although the insect may attempt to feed on Cupressaceae or Taxaceae, *F. externa* appears unable to sustain populations on plants from these families.

*Fiorinia externa*, like other Diaspididae, is recognized as a parasite of its host plants (58). Females are incapable of directed long-distance movements, and directional movements are constrained by the walking capacity of crawlers, potentially <1 m (59). Further, once a crawler establishes a feeding site, it cannot retract its stylets to continue host searching (25). Thus, behavior plays little role in host choice for this species unlike many other oligophagous/polyphagous insects. The limited dispersal abilities of this insect put it under strong evolutionary pressure to attempt feeding on a potential host and not kill it until densities are high. Similarly, limited dispersal has contributed to the rapid micro-evolution of *F. externa* to different host species (60) and potentially to environmental tolerances (61). Demic adaptations to hosts are not so strong as to preclude colonization of new hosts (59).

The risk that *F. externa* poses to regions beyond the range of *Tsuga* spp. is highly uncertain. Pest risk, in this context, is a function of the likelihood that *F. externa* might invade a new area and the extent of damages it might cause once there. If introduced to new areas on cut foliage, the insect may face significant challenges to locate live host material and withstand local climatic conditions. The arrival of *F. externa* on propagative hosts would thus seem to be the riskier pathway. Despite being present in the United States for >100 years, the circumstances that might lead *F. externa* to kill otherwise healthy trees are only coarsely characterized. Widespread tree mortality from *Fiorinia externa* has never been documented, even on preferred hosts. Evidence from prior pest activity and sentinel trees (62) indicates that although future host mortality from *F. externa* is possible, extensive host mortality is unlikely.

## Author contributions

RV: Writing – original draft, Writing – review & editing. AA: Writing – review & editing. BA: Writing – review & editing. RJ: Writing – review & editing. TP: Writing – review & editing.

## References

[1] Preisser EL Oten KLF Hain FP. Hemlock wooly adelgid in the eastern United States: what have we learned? Southeastern Nat (2014) 13(Special Issue 6):1–15.

[2] Sasscer ER. The genus Fiorinia in the United States. Technical series No. 16. Part V Howard LO, editors. Washington, D.C: Government Printing Office (1912).

[3] Davidson JA McComb CW. Notes on the biology and control of *Fiorinia externa* Ferris. J Econ Entomol (1958) 51(3):405–6.

[4] Bray DF. *Fiorinia* hemlock scale. Sci Tree Topics (1958) 2(5):11.

[5] Wallner WE. Biology and control of Fiorinia hemlock scale *Fiorinia externa* Ferris. Ithaca, NY: Cornell University (1965).

[6] Stimmel JF. Seasonal history and occurrence of *Fiorinia externa* in Pennsylvania (Homoptera: Diaspididae). Proc Entomol Soc Wash (1980) 82(4):700–6.

[7] McClure MS. Controlling hemlock scales with least environmental impact. Bulletin 844. New Haven, CT: The Connecticut Agricultural Experiment Station (1987). 8 p.

[8] USDA. Elongate hemlock scale, Fiorinia externa Ferris: Alien forest pest explorer - species map. USDA Forest Service, Northern Research Station and Forest Health Protection (2019). Available at: https://www.fs.usda.gov/nrs/tools/afpe/maps/pdf/EHS.pdf.

[9] Hudgins EJ Liebhold AM Leung B. Predicting the spread of all invasive forest pests in the United States. Ecol Letters (2017) 20(4):426–35. doi: 10.1111/ele.12741 28176497

[10] McClure MS. Importance of weather to the distribution and abundance of introduced adelgid and scale insects. Agric For Meteorol (1989) 47(2-4):291–302. doi: 10.1016/0168-1923(89)90101-9

[11] Ferris GF. Atlas of scale insects of North America. Stanford, CA: Stanford University Press (1942).

[12] Takagi S. Discovery of *Fiorinia externa* Ferris in Japan (Homoptera: Coccoidea). Insecta Matsumurana (1963) 26(2):115–7.

[13] Wei J Zhang B Feng J. Two new species of *Fiorinia* Targioni-Tozzetti (Hemiptera: Coccoidea: Diaspididae) from China. Zootaxa (2013) 3641(1):092–100. doi: 10.11646/zootaxa.3641.1.10 26287071

[14] Tao C-c. List of Coccoidea (Homoptera) of China. Wufeng, Taiching, Taiwan R.O.C: Taiwan Agricultural Research Institute Special Publication No. 78 (1999). 176 p.

[15] Van Driesche R Reardon R Montgomery M Cowles R Abell K Nunn C. Classical biological control of the elongate hemlock scale, *Fiorinia externa*: 2004 activities. In: Onken B Reardon R, editors. Third symposium on hemlock wooly adelgid in the eastern United States. Asheville, NC: U.S. Department of Agriculture, Forest Service, Forest Health Technology Enterprise Team (2005). p. 135–44.

[16] Williams DJ. *Fiorinia externa* Ferris (Hemiptera: Diaspididae) found in Surrey infesting *Abies koreana* . Entomologist’s Gazette (1988) 39(2):151–2.

[17] Malumphy C. First incursion of crown scale *Fiorinia coronata* Williams & Watson (Hemiptera: Diaspididae) in England, with a review of *Fiorinia* species detected in Britian and a key to their identification. Entomologist’s Gazette. (2013) 64:269–76.

[18] Kosztarab M. Scale insects of northeastern North America: Identification, biology, and distribution. Martinsville, VA: Virginia Museum of Natural History (1996). 650 p.

[19] Leathers J. California Pest Rating for Fiorinia externa Ferris: elongate hemlock scale (Hemiptera: Diaspididae) Pest Rating A. Sacramento, CA: California Department of Food and Agriculture (2016).

[20] Petrice TR Poland TM Ravlin FW. Proceedings of the 7th North American Forest Insect Work Conference. Elongate hemlock scale in Michigan: initial assessment of distribution, impacts, and natural enemies. In: Arango RA Pureswaran DS, editors. Proceedings of the 7th North American Forest Insect Work Conference; Virtual: U.S. Department of Agriculture, Forest Health Assessment and Applied Sciences Team (Washington, DC: U.S. Department of Agriculture, Forest Health Assessment and Applied Sciences Team) (2022). p. 95–7.

[21] Ambourn A Shimek S. Proceedings of the 7th North American Forest Insect Work Conference. The scale that stole Christmas. In: Arango RA Pureswaran DS, editors. Proceedings of the 7th North American Forest Insect Work Conference; Virtual: U.S. Department of Agriculture, Forest Health Assessment and Applied Sciences Team (2022). p. 136–7.

[22] Darr MN Coyle DR Jetton RM. Arthropod and disease management in Fraser fir (Pinales: Pinaceae) Christmas trees in the southeastern United States. Washington, D.C., J Integr Pest Manage (2022) 13(1):17. doi: 10.1093/jipm/pmac001

[23] Stocks IC. Armored scale (Hemiptera: Diaspididae) pests on *Abies fraseri* (Pinaceae) Christmas trees imported into Florida. Florida Entomol (2016) 99(4):785–7. doi: 10.1653/024.099.0435

[24] Ahmed MZ Moore MR Rohrig EA McKenzie CL Liu D Feng J. Taxonomic and identification review of adventive *Fiorinia* Targioni Tozzetti (Hemiptera, Coccomorpha, Diaspididae) of the United States. ZooKeys (2021) 1065:141–203. doi: 10.3897/zookeys.1065.69171 36452345 PMC9616077

[25] Beardsley JW Gonzalez RH. Biology and ecology of armored scales. Annu Rev Entomol (1975) 20:47–73. doi: 10.1146/annurev.en.20.010175.000403 1090245

[26] Koch FH. Considerations regarding species distribution models for forest insects. Agric For Entomol (2021) 23(4):393–9. doi: 10.1111/afe.12458

[27] García Morales M Denno BD Miller DR Miller GL Ben-Dov Y Hardy NB. ScaleNet: A literature-based model of scale insect biology and systematics (2016). Available at: http://scalenet.info. 10.1093/database/bav118 PMC4747323 26861659

[28] Garrett WT Langford GS. Seasonal life cycle of *Fiorinia externa* in Maryland. J Econ Entomol (1969) 62(5):1221–2. doi: 10.1093/jee/62.5.1221

[29] McClure MS. Armored scale insects: Their biology, natural enemies and control (Volume A). Rosen D, editor. New York: Elsevier (1990).

[30] Talerico RL McComb CW Garrett WT. Forest Pest Leaflet 107: Fiorinia externa Ferris, a scale insect of hemlock. Washington, DC: U.S. Department of Agriculture, Forest Service (1971).

[31] McClure MS. Resurgence of scale, *Fiorinia* externa (Homoptera: Diaspididae), on hemlock following insecticide application. Environ Entomol (1977) 6(3):480–4. doi: 10.1093/ee/6.3.480

[32] McClure MS. Population dynamics of Japanese hemlock scales: a comparison of endemic and exotic communities. Ecology (1986) 67(5):1411–21. doi: 10.2307/1938696

[33] Stoetzel MB. Scale-cover formation in Diaspididae (Homoptera: Coccoidea). Proc Entomol Soc Wash (1976) 78(3):323–32.

[34] McClure MS. Spatial and seasonal distribution of disseminating stages of *Fiorinia* externa (Homoptera, Diaspididae) and natural enemies in a hemlock forest. Environ Entomol (1979) 8(5):869–73. doi: 10.1093/ee/8.5.869

[35] McClure MS Fergione MB. *Fiorinia externa* and *Tsugaspidiotus tsugae* (Homoptera: Diaspididae): distribution, abundance, and new hosts of two destructive scale insects of eastern hemlock in Connecticut. Environ Entomol (1977) 6(6):807–11. doi: 10.1093/ee/6.6.807

[36] McClure MS. Foliar nitrogen: a basis for host suitability for elongate hemlock scale, *Fiorinia* externa (Homoptera: Diaspididae). Ecology (1980) 61(1):72–9. doi: 10.2307/1937157

[37] Dale AG Birdsell T Sidebottom J. Evaluating the invasive potential of an exotic scale insect associated with annual Christmas tree harvest and distribution in the southeastern U.S. Trees Forests People (2020) 2:100013. doi: 10.1016/j.tfp.2020.100013

[38] Weston PA Harper RW. Potential of *Tsuga* spp. from western North America and Asia as replacements for eastern hemlock (*Tsuga canadensis*). Arboricult Urban Forest (2009) 35(1):5–9.

[39] Wallner WE. A field test with insecticides to control the scale *Fiorinia externa* on Canadian hemlock. J Econ Entomol (1962) 55(5):798–9. doi: 10.1093/jee/55.5.798a

[40] Garrett WT Langford GS. Control of *Fiorinia externa* on hemlock in Maryland. J Econ Entomol (1969) 62:1449–50. doi: 10.1093/jee/62.6.1449

[41] Felt EP. Observations on shade tree insects. J Econ Entomol (1933) 26(1):45–51. doi: 10.1093/jee/26.1.45

[42] Murakami Y. A review of biology and ecology of diaspine scales in Japan (Homoptera: Coccoidea). Mushi (1970) 43(25):65–114.

[43] Schaeffer RN Wang Z Thornber CS Preisser EL Orians CM. Two invasive herbivores on a shared host: patterns and consequences of phytohormone induction. Oecologia (2018) 186:973–82. doi: 10.1007/s00442-018-4063-0 29362885

[44] McClure MS. Dispersal of the scale *Fiorinia* externa (Homoptera: Diaspididae) and effects of edaphic factors on its establishment on hemlock. Environ Entomol (1977) 6(4):539–44. doi: 10.1093/ee/6.4.539

[45] Miller DR Miller GL Hodges GS Davidson JA. Introduced scale insects (Hemiptera: Coccoidea) of the United States and their impact on U.S. agriculture. Proc Entomol Soc Wash (2005) 107(1):123–59.

[46] Zahradnik J. Forests. In: Rosen D, editor. Armored scale insects: Their biology, natural enemies and control. Volume B. New York: Elsevier (1990). p. 633–7.

[47] Mech AM Thomas KA Marsico TD Herms DA Allen CR Ayres MP. Evolutionary history predicts high-impact invasions by herbivorous insects. Ecol Evol (2019) 9(21):12216–30. doi: 10.1002/ece3.5709 PMC6854116 31832155

[48] McClure MS. Competition between herbivores and increased resource heterogeneity. In: Denno RF McClure MS, editors. Variable plants and herbivores in natural and managed systems. New York: Academic Press (1983). p. 125–53.

[49] Miller-Pierce MR Orwig DA Preisser E. Effects of hemlock woolly adelgid and elongate hemlock scale on eastern hemlock growth and foliar chemistry. Environ Entomol (2010) 39(2):513–9. doi: 10.1603/EN09298 20388282

[50] McClure MS. The elongate hemlock scale, *Fiorinia externa* Ferris (Homoptera: Diaspididae): a new look at an old nemesis. In: Oken B,RR Lashomb J, editors. Symposium on the hemlock woolly adegid in eastern North America. East Brunswick, NJ: New Jersey Agricultural Experiment Station, Rutgers (2002). p. 248–53.

[51] Johnson WT Lyon HH. Insects that feed on trees and shrubs. Ithaca, NY: Cornell University Press (1991). 560 p.

[52] Preisser EL Lodge AG Orwig DA Elkinton JS. Range expansion and population dynamics of co-occurring invasive herbivores. Biol Invasions (2008) 10(2):201–13. doi: 10.1007/s10530-007-9123-z

[53] Preisser E Elkinton JS. Exploitative competition between invasive herbivores benefits a native host plant. Ecology (2008) 89(10):2671–7. doi: 10.1890/08-0299.1 18959304

[54] Gonda-King L Radville L Preisser EL. False ring formation in eastern hemlock branches: impacts of hemlock woolly adelgid and elongate hemlock scale. Environ Entomol (2012) 41(3):523–31. doi: 10.1603/EN11227 22732610

[55] Miller-Pierce MR Preisser EL. Asymmetric priority effects influence the success of invasive forest insects. Ecol Entomol (2012) 37(5):350–8. doi: 10.1111/j.1365-2311.2012.01371.x

[56] Gomez S Gonda-King L Orians CM Preisser EL. Competitor avoidance drives within-host feeding site selection in a passively dispersed herbivore. Ecol Entomol (2014) 39(1):10–6. doi: 10.1111/een.12059

[57] Ran JH Shen TT Wu H Gong X Wang XQ. Phylogeny and evolutionary history of Pinaceae updated by transcriptomic analysis. Mol Phylogenet Evol (2018) 129:106–16. doi: 10.1016/j.ympev.2018.08.011 30153503

[58] Morse GE Normark BB. A molecular phylogenetic study of armoured scale insects (Hemiptera: Diaspididae). Syst Entomol (2006) 31(2):338–49. doi: 10.1111/j.1365-3113.2005.00316.x

[59] Hanks LM Denno RF. The role of demic adapatation in colonization and spread of scale insect populations. In: Kim KC McPheron BA, editors. Evolution of insect pests: Patterns of variation. New York: John Wiley & Sons (1993). p. 393–412.

[60] McClure MS. Reproduction and adpations of exotic hemlock scales (Homptera: Diaspididae) on their new and native hosts. Environ Entomol (1983) 12:1811–5. doi: 10.1093/ee/12.6.1811

[61] Preisser EL Elkinton JS Abell K. Evolution of increased cold tolerance during range expansion of the elongate hemlock scale *Fiorinia externa* Ferris (Hemiptera: Diaspididae). Ecol Entomol (2008) 33(6):709–15. doi: 10.1111/j.1365-2311.2008.01021.x

[62] Raffa KF Brockerhoff EG Gregoire JC Hamelin RC Liebhold AM Santini A. Approaches to forecasting damage by invasive forest insects and pathogens: a cross-assessment. Bioscience (2023) 73(2):85–111. doi: 10.1093/biosci/biac108

